# Effects of a beaver dam on the benthic copepod assemblage of a Mediterranean river

**DOI:** 10.1038/s41598-024-59456-y

**Published:** 2024-04-18

**Authors:** T. Di Lorenzo, A. Tabilio Di Camillo, E. Mori, A. Viviano, G. Mazza, A. Pontalti, M. Rogora, B. Fiasca, M. Di Cicco, D. M. P. Galassi

**Affiliations:** 1 National Research Council of Italy, Research Institute on Terrestrial Ecosystems (CN-IRET), Florence, Italy; 2 NBFC (National Biodiversity Future Center), 90133 Palermo, Italy; 3 Department of Life, Health and Environmental Sciences, University of L’Aquila, L’Aquila, Italy; 4 CREA Research Centre for Plant Protection and Certification (CREA‑DC), Florence, Italy; 5 National Research Council of Italy, Water Research Institute (CNR-IRSA), Verbania Pallanza, Italy

**Keywords:** *Castor fiber*, Functional traits, Meiofauna, Rodentia, Semi-lentic, Lotic, Biodiversity, Ecology, Freshwater ecology

## Abstract

As known “ecosystem engineers”, beavers influence river hydrology, geomorphology, biochemistry, and biological assemblages. However, there is a lack of research regarding the effects of beaver activities on freshwater meiofauna. In this study, we investigated the taxonomic and functional composition of the benthic copepod assemblage of a segment of the Tiber River (Italy) where a beaver dam, created about 7 weeks before our survey, had formed a semi-lentic habitat upstream and a lotic habitat downstream of the dam. We also analyzed the copepod assemblage before and after a flood event that destroyed the beaver dam, providing a unique opportunity to observe changes in a naturally reversing scenario. Our analyses revealed that, while the taxonomic composition and functional traits of the copepod assemblage remained largely unchanged across the recently formed semi-lentic and lotic habitats, substantial differences were evident between the dammed and undammed states. The dammed state showed lower copepod abundances, biomass, and functionality than the undammed one. These results highlight the role of beaver dams in changing the composition and functionality of meiofaunal assemblages offering insights into the dynamic interactions within aquatic ecosystems.

## Introduction

The Eurasian beaver (*Castor fiber*) is a rodent that inhabits woodlands and freshwater habitats in a wide range of environments across Europe and Asia^1^. Like its North American counterpart *C. canadensis*, the Eurasian beaver has suffered historical persecution by humans, leading to a decline in distribution and genetic diversity^2^. However, conservation efforts, including legal protection, hunting regulation, reintroductions, and natural recolonization, have allowed the species to recover much of its former range^1^. The Italian peninsula was once home to beavers during the Pleistocene and Holocene, but the species was extirpated in the 16th or 17th century^3,4^. In October 2018, a single beaver individual was spotted in Tarvisio, and its presence was later confirmed in Val Pusteria, where, currently, two individuals may occur^5^. In early 2021, evidence of beaver activity was found in two river basins in Tuscany and Umbria regions, indicating the presence of a breeding resident population^5,6^. Subsequent surveys have revealed signs of beaver activity in other regions of Italy^7^. Genetic analysis has confirmed the beaver species in Italy as *C. fiber*, and the populations clustered within the variability of the Western clade, including individuals from Central Europe^6^. Afterwards, in 2023, other specimens were spotted in other regions of Central and Southern Italy^4^.

The effects of beavers on water quality and biodiversity of lotic habitats vary across regions and ecosystems^8^. Most studies have focused on local scales, making it arduous to draw comprehensive conclusions about the overall effects of beavers^8^, which encompass impacts on river hydrology, geomorphology, biochemistry, and biological assemblages^9^. Their dams reduce water current and raise the water level, creating ponds upstream of the dam that allow beavers to construct their den or lodge^10^. Locally, these semi-lentic habitats promote sediment storage and flood attenuation^11^ and act as filters for pollutants and sinks for nutrients^9^. The creation of canals promotes hydrological connectivity, but other beaver activities, such as burrowing and vegetation removal, may increase bank erosion^11^. The construction of dams increases the surface of intertidal zones, which benefits riparian plant associations that are often degraded due to land use^9^. Beavers’ herbivory of preferred plants increases the density of non-preferred species, thus altering the plant community’s species composition^12^. Ponds created by beaver dams increase mammalian species richness, especially for semiaquatic mammals like otters and moose, as well as potential breeding sites for wading birds and saproxylic beetles^13^. The activities of beavers create a mosaic landscape of aquatic environments that locally increase the diversity of invertebrates^14^. However, the increase of fine sediment in ponds may decrease the density of rheophilic invertebrate taxa in naturally pebble-dominant riverbeds^14^. Ponds can serve as refuges for riparian organisms during droughts and increase the diversity and biomass of aquatic insects living in soft organic-laden sediments^15^. Ponds also favor plants and algae, which may increase the biomass of grazing insects and crustaceans^15^. Reduced silt, low flow rates, and an increased abundance of invertebrates can create important spawning and feeding points for salmonids downstream of the dam^16^. The local increase in biomass and diversity of macroinvertebrates inside or near beaver ponds also facilitates the presence and abundance of several species of amphibians and reptiles^17^. Environmental engineering by beavers is of great ecological significance also for small benthic organisms. However, there is a critical knowledge gap regarding the effects of beavers’ activities on meiofauna^8^.

Meiofauna are small organisms ranging from 36 to 1000 μm that inhabit freshwater, brackish, and marine habitats and play an important role in maintaining ecosystem functioning^18^. The distribution of lotic meiofauna is influenced by environmental factors, mainly water flow and granulometry^19^, which, in turn, are affected by beavers’ activity. Copepods (Crustacea Copepoda) are among the most abundant and species rich taxa in lotic meiofaunal crustacean assemblages^20^. However, the effects of beavers on lotic meiofauna are unknown. To fill this knowledge gap, we surveyed the benthic copepod assemblage of a segment of the Tiber River in Italy, where a beaver dam resulted in the formation of a semi-lentic habitat upstream and a lotic habitat downstream of the dam. The dam in question was relatively new, having been constructed in December 2021, about 7 weeks prior to our survey, as indicated by reports from local fishermen. It was also small and featured two openings that allowed for some water flow. We hypothesized there were differences in the taxonomic composition of the copepod assemblage between the two habitats (semi-lentic *vs* lotic). We postulated a higher abundance of benthic copepods in the semi-lentic habitat, attributed to more stable sediment conditions and greater availability of organic matter, compared to the lotic habitat. We also predicted that the life history and functional traits of the copepod assemblages, including their feeding habits and reproductive strategies, would vary substantially between the semi-lentic and lotic segments. At the end of September 2022, a flood swept away the dam. This event provided the opportunity to explore whether and how these effects persisted or changed after the dam was removed.

## Materials and methods

### Study area

**Figure 1:**
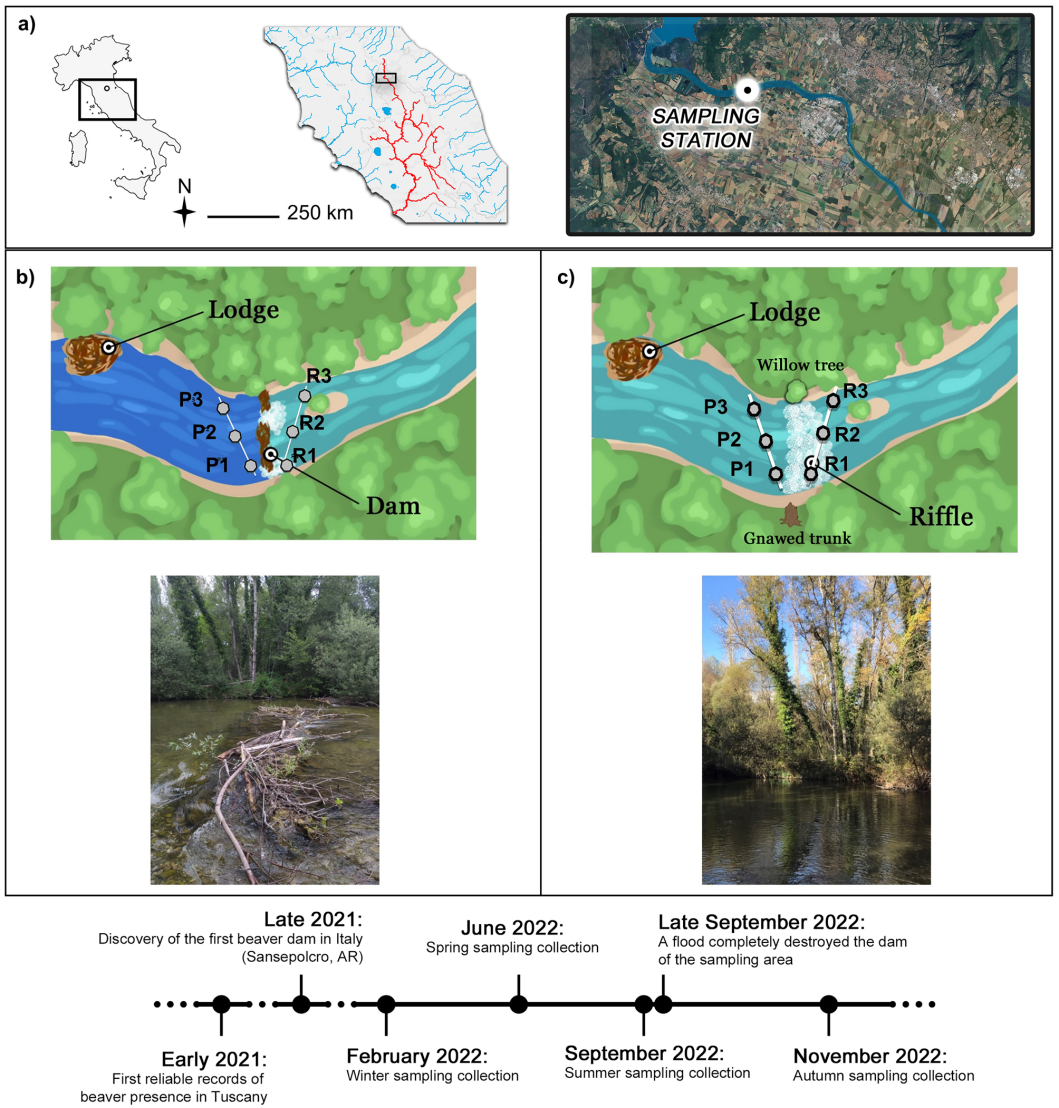
Study area and sampling station. (**a**) Sansepolcro sampling station, Tiber River in Tuscany (Italy); (**b**) transects oblique to the river channel upstream (semi-lentic habitat) and downstream (lotic habitat) of the beaver dam and location of the beaver lodge; (**c**) transects oblique to the river channel in the undammed state (after the dam removal by the flood event at the end of September 2022) and location of the beaver lodge. P1, P2, P3: samples in the semi-lentic habitat; R1, R2, R3: samples in the lotic habitat. Bottom: timeline showing the time setting of the study. The satellite image was obtained from Google Earth Pro vs. 10.46.0.2.

We carried out the study in the headwaters of the Tiber River (406 r.km), at the sampling station of Sansepolcro (coordinates, WGS84: Lat. 43.5710, Long. 12.0859; 300 m above sea level; climate: warm temperate/humid Mediterranean; mean annual precipitation: 946 mm; Fig. 1), in Tuscany (Italy). The station is classified as a Salmonid Zone pursuant to Italian regulation; it is characterized by a low discharge, rapid flow, stony bottom, and a fish fauna dominated by trout and gobies. The 100 m-wide riparian zone is well-preserved and heavily forested with flood-resistant species like willows (*Salix* spp.), poplars (*Populus* spp.), and alders (*Alnus* spp.), as well as invasive black locusts (*Robinia pseudoacacia*). In Sansepolcro, the catchment basin of the Tiber River is predominantly utilized for urban and agricultural purposes. Two km upstream of the sampling station, there is a small protected riparian area (Golena del Tevere protected area, 175 ha).

### Sampling surveys

The beaver dam (10 m in length; about 40–60 cm in height; made of small-to-medium-sized logs/sticks, mostly willows and poplars) had been erected on top of a riffle, and there were two openings, one at the far end of the river channel and a second in the left middle bank, so that the dam did not wholly impede the flow (Fig. 1). Despite the two openings, the dam created a pond upstream (hereafter referred to as “semi-lentic habitat”; Fig. 1), as opposed to a lotic stretch downstream (hereafter referred to as “lotic habitat”; Fig. 1). The beaver lodge was located on the left shore of the pond, roughly a dozen meters upstream of the dam. We performed three sampling surveys: in February, June, and September 2022. In each habitat, we collected samples (P1, P2, P3 in the semi-lentic habitat and R1, R2, R3 in the lotic habitat) along two transects oblique to the river channel (Fig. 1). Using some large poplars and gnawed trunks as landmarks, we located the starting points of the transects at 50 cm from the dam and the ending points at 250 cm (Fig. 1) to encompass major differences observed in sediment composition and water depth. Overall, we took a total of 18 biological samples (9 upstream and 9 downstream of the dam) from February to September 2022. The dam was swept away by a flood at the end of September 2022, 10 days after our third sampling survey. The flood modified the morphology of the sampling station (Fig. 1). We left the system to recover for 45 days after the flood. In mid-November 2022, we carried out a final sampling survey. Using the previous landmarks, we estimated the position of the transects as in the previous sampling surveys and took six additional samples bringing the total to 24 samples.

We used a standard methodology of kick sampling consisting in disturbing the substrate on the riverbed in an area of about 625 cm^2^ by foot up to 5 cm for 30 s^21^. After substrate disturbing, we collected the suspended sediment and the dislodged meiofauna using a hand net with a mesh of 60 μm, quickly dragging it over the disturbed area, opposite the flow^21^. We closed the net underwater before withdrawing it to the surface to avoid filtering the water column. Once collected, we bottled the samples and preserved them in a 70% ethanol solution. Given the inherent limitations of field sampling, maintaining a consistent depth of 5 cm was not always feasible. To ensure uniformity and comparability, we standardized the samples in the laboratory. For this purpose, we sorted through the samples and collected the initial 150 meiobenthic specimens, which included copepods. We used a glass pipette and a Leica M80 stereomicroscope set at 16 × magnification for this task. We identified the specimens at the species/subspecies level using taxonomic keys^22–26^ and additional specialized literature. Life history traits were examined by attributing each copepod individual to one of the following stages: ovigerous female, non-ovigerous female, male, copepodid, and nauplius. We evaluated five functional traits, each described by two or more categories, as following: body shape (cylindrical, pyriform), locomotion (burrower, interstitial endobenthic, swimmer), diet (fine sediments + microorganisms, fine sediments + microorganisms + living microinvertebrates, living microinvertebrates, living microphytes, omnivorous), feeding habits (deposit feeder, deposit feeder + scraper, deposit feeder + opportunist, predator, grazer), and thermal preference (highly eurythermal, eurythermal, moderately stenothermal, stenothermal). The body shape of organisms can be regarded as a functional trait as it embodies adaptations to distinct ecological niches, feeding strategies, and behaviors, as well as physiological adjustments concerning thermoregulation, buoyancy control, and predator evasion^21,27^. Body shape was assessed directly in the laboratory, while the remaining traits were attributed to each specimen based on previous studies^27,28^ and specialized literature^23,24^. We obtained the trait profile of each sample by weighing traits’ abundances in the sample and rescaling them to 0–100^27,29^. Finally, we measured each copepod individual by taking a picture with a LEICA M80 stereomicroscope equipped with an integrated camera and using the LAS program (Leica Application Suite, version 4.7.1). We used the equation in Reiss & Schmid-Araya^30^ to convert the body length and width (in mm) into dry carbon to estimate the biomass.

We assessed the granulometry of each sample by visually estimating the composition of the stream bed within a 45 × 45 cm square before kick sampling. Following the Udden-Wentworth grain-size scale^31^, inorganic sediment was apportioned into boulders (> 256 mm), large cobbles (256–131 mm), small cobbles (130–65 mm), pebbles (64–17 mm), gravels (16–4 mm), sand (3–0.063 mm), and silt (< 0.063 mm). We also measured, in the middle of the riverbed, in both the semi-lentic and lotic habitats, temperature, pH, conductivity, and dissolved oxygen by using the multiparameter probe YSI 6000 (YSI Inc., Yellow Springs, OH, USA). Finally, we collected 2 L of water at the central point of each transect at each date to analyze the following parameters: total alkalinity, chloride, sulfate, nitrate, ammonium, calcium, magnesium, sodium, potassium, reactive and total phosphorus, reactive silica, and total organic carbon. All the analyses were performed in the laboratory of CNR-IRSA in Verbania according to standard methods for freshwater samples.

### Data analysis

All statistical analyses were performed using the softwares PRIMER v7^32^ and R^33^. To perform the RQL and fourth-corner analyses, we employed *ade4* and *vegan* packages.

We used Permutational Multivariate Analysis of Variance (PERMANOVA) to assess variations in granulometry, physical and chemical properties, and taxonomic and functional composition of the copepod assemblage between the semi-lentic and lotic habitats and between the dammed and undammed states of the sampling station. We use two fixed factors: Habitat (two levels: semi-lentic and lotic) and State (two levels: dammed and undammed). Month was incorporated as a random factor (four levels: February, June, September, and November) and nested within the State factor. PERMANOVA sits on one assumption only, that the samples are exchangeable under a true null hypothesis^34^. Since samples having different dispersions in different groups are not exchangeable, we used the PERMDISP routine prior to PERMANOVAs to account for the potential heterogeneity of the variances within each factor^34^. We used similarity matrices based on Euclidean distances of raw data for granulometry and normalized data for the physical and chemical variables. We used Bray–Curtis similarity matrices of log(x + 1) transformed abundances and raw trait data. We set the significance level (α) equal to 0.05.

We evaluated the biological sampling’s thoroughness by looking at the rising copepod species richness (S) as samples were successively pooled. We used one parametric (Michaelis–Menten) and six non-parametric (Chao1, Chao2, Jackknife1, Jackknife2, Unbiased Gradient Estimator, and Bootstrap) estimators to obtain curves of S increasing with sample size^35^. We set value estimations by using 999 randomizations without replacement. We computed indices of taxonomic (Margalef’s—*d*, Pielou’s—*J*, Shannon’s—*H,* and Simpson’s—*1-λ*) and functional (Functional Richness -*FRic*, Functional Evenness—*FEve*, Functional Divergence—*FDiv*, Functional Dispersion—*FDis*) diversity per each sample. All diversity indices were computed using the *vegan* package in R.

We also conducted SIMPER analyses^35^ to decompose the average dissimilarity between sample groups, quantifying the individual species’ or taxa’s percentage contributions to the observed differences. We used distance-based linear models (DistLM)^36^ to examine potential linear relationships between taxonomic composition and environmental variables. We applied the BEST selection procedure, which looks at the selection criterion for all possible combinations of predictor variables. We then used the AIC (Akaike Information Criterion) criterion^37^ to choose the best model and R^2^ to evaluate its fit. We evaluated the fit of the models using p-values obtained from 999 permutations, and we considered significance to be at α = 0.05. To explore the relationships between species traits and environmental conditions, we combined the RQL^38^ and fourth-corner analyses^39^. The RQL analysis integrates the relationships between three matrices (species traits, environmental variables, and species abundance) to discern how specific species traits are related to environmental conditions^38^. We used a Correspondence Analysis for the abundance matrix, while Principal Component Analyses were used for the environmental and trait ones. Afterward, we assessed the strength and significance of the pairwise correlations between traits and environmental variables with the aid of the fourth-corner analysis where the statistical significance was corrected by Bonferroni adjustment and set at α < 0.001.

## Results

### Environmental condition

The percentages of granulometric classes and the values of physical and chemical parameters are reported in Supplementary Tables S1 and S2, respectively. Mean values are reported in Table 1. PERMANOVAs indicated significant differences in the granulometric composition between the semi-lentic and lotic habitats (Pseudo-F_1,23_ = 16.04, p-value = 0.020) and between the dammed and undammed states (Pseudo-F_1,23_ = 18.64, p-value = 0.03). Specifically, the samples of the semi-lentic habitat contained more gravel and sand, while those collected from the lotic habitat were richer in small cobbles and pebbles (Table 1). The samples of the dammed state had a higher content in gravel, silt and sand than those collected in the undammed state, which were richer in small cobbles and pebbles (Table 1).

**Table 1:** Average values of granulometry and physical and chemical variables of the Tiber River at the Sansepolcro station.

Variable	µSL	µLR	µD	µUN
Granulometry				
Boulders > 256 mm (%)	0.0	0.0	0.0	0.0
Large cobbles 256–131 mm (%)	0.0	0.0	0.0	0.0
Small cobbles 130–65 mm (%)	0.0	30.0	15.0	15.0
Pebbles 64–17 mm (%)	20.0	43.6	31.3	85.0
Gravel 16–4 mm (%)	62.6	20.0	41.3	13.7
Sand 3–0.063 mm (%)	8.0	3.0	5.5	1.2
Silt < 0.063 mm (%)	7.2	4.3	5.8	0.2
Physical and chemical				
O_2_ (mg L^−1^)	13.23	13.67	13.45	13.50
T (°C)	12.60	12.60	12.50	14.71
pH	7.92	8.25	8.09	8.27
EC (μS cm^−1^ at 25 °C)	384.73	383.80	384.27	364.55
Tot. Alk. (meq L^−1^)	3.65	3.65	3.65	3.35
Cl (mg L^−1^)	9.87	9.87	9.87	10.25
SO_4_ (mg L^−1^)	29.27	29.67	29.47	28.95
N-NO_3_ (μg L^−1^)	170	192	181	104
N-NH_4_ (μg L^−1^)	13	28	21	14
Ca (mg L^−1^)	58.87	59.90	59.38	54.00
Mg (mg L^−1^)	13.20	12.93	13.07	12.40
Na (mg L^−1^)	10.43	10.13	10.27	10.70
K (mg L^-1^)	1.52	1.59	1.55	1.9
RP (μg P L^−1^)	3	4	4	3
TP (μg P L^−1^)	7	7	7	6
SiO_2_ (mg Si L^−1^)	1.98	2.01	1.99	1.35
TOC (mg C L^−1^)	1.79	1.89	1.84	2.01

Mean values in the semi-lentic (µSL) and lotic (µLR) habitats; mean values of the dammed (µD) and undammed (µUN) states of the Tiber River. *T* Temperature, *EC* Electrical conductivity at 25 °C, *Tot. Alk*. Total alkalinity, *RP* Reactive phosphorus, *TP* Total phosphorus, *TOC* Total organic carbon.

The waters of the Tiber River at Sansepolcro sampling station were characterized by circumneutral or basic pH and a moderate solute content (Table 1), with calcium and bicarbonate (represented by total alkalinity) dominating among cations and anions, respectively (about 32 and 40% of the total ionic content). According to the results of the nutrient analyses, the water of Tiber River at the sampling station proved to be of high quality: specifically, the concentrations of nutrients that can be related to anthropogenic impact, such as ammonium and phosphorus compounds were low and, in some cases, close to the minimum detectable level. As an example, reactive and total phosphorus concentrations were indicative of oligotrophic conditions (Table 1). Also, the content of organic matter was rather low according to the TOC concentrations (Table 1). The chemical and physical conditions did not show any significant differences between the two habitats (Pseudo-F_1,7_ = 0.32, p-value = 0.114) or between the dammed and undammed states (Pseudo-F_1,7_ = 0.69, p-value = 0.062).

### Taxonomic composition

We reported the average values of taxonomic data in Table 2. Overall, we collected 1215 copepod individuals from 10 species (Supplementary Table S3). The most common species was *Nitocra hibernica* (Brady, 1880), which accounted for 48% of the total, followed by *Attheyella (Attheyella) crassa* (Sars G.O., 1863), *Bryocamptus (Echinocamptus) echinatus* (Mrázek, 1893), and *Bryocamptus (Bryocamptus) pygmaeus* (Sars G.O., 1863), which represented 19%, 18%, and 4.4% respectively. The remaining six species (*Canthocamptus (Canthocamptus) staphylinus* (Jurine, 1820), *Epactophanes richardi* Mrázek, 1893; *Acanthocyclops vernalis* (Fischer, 1853), *Macrocyclops albidus* (Jurine, 1820), *Microcyclops varicans* (Sars G.O., 1863), *Paracyclops fimbriatus* (Fischer, 1853)) accounted for less than 2% each. All estimators suggested that we comprehensively sampled the copepod diversity in the analyzed segment of the Tiber River (see Supplementary Fig. S1).

**Table 2:** Average values of taxonomic and functional data in the Tiber River at the Sansepolcro station.

Variable	µSL	µLR	µD	µUN
Taxonomic diversity				
S	4	4	4	4
d	0.6	0.6	0.6	0.5
J	0.6	0.5	0.6	0.4
H	0.7	0.6	0.7	0.5
1−λ	0.4	0.4	0.5	0.3
Functional diversity				
FRic	8.3	7.1	5.8	13.0
FEve	0.6	0.5	0.7	0.4
FDiv	0.8	0.8	0.7	0.9
FDis	1.8	1.9	1.9	1.5
Functional traits				
Biomass μg C	20.89	22.44	22.44	71.33
A—Adults (%)	55.00	62.45	58.73	61.15
C—Juveniles (%)	45.00	37.55	41.27	38.85
F—Females (%)	72.06	72.45	71.70	74.28
M—Males (%)	27.94	27.55	28.30	25.72
OF—Ovigerous females (%)	28.61	19.28	23.95	4.90
NOF—non ovigerous females (%)	71.39	80.72	76.05	95.10
N—Nauplii (%)	7.05	8.79	7.92	0.81
Body shape				
Cyl—Cylindrical (%) [**Acr,Ave,Bec,Cst,Nhi,Eri,Bpy**]	93.0	85.1	89.1	96.7
Pyr – Pyriform (%) [**Mal,Mva,Pfi**]	7.0	14.9	10.9	3.3
Locomotion				
Bur—Burrower (%) [**Acr,Cst,Nhi.Pfi**]	47.7	38.7	43.2	89.0
Int—Interstitial (%) [**Bec,Bpy,Eri**]	35.1	50.9	43.0	10.9
Swi—Swimmer (%) [**Ave,Mal,Mva**]	17.2	10.4	13.8	0.1
Diet				
FS_M—Fine sed. + microorg. (%) [**Acr,Bec,Cst,Nhi,Eri,Bpy**]	82.8	87.5	85.2	95.2
FS_M_Lmin—Fine sed. + microor. + living microinv. (%) [**Mal**]	2.0	4.0	3.0	1.1
Lmin—Living microinv. (%) [**Ave**]	10.3	1.2	5.7	0.0
Lmic—Living microp. (%) [**Mva**]	5.0	1.6	3.3	0.0
O—Omnivore (%) [**Pfi**]	0.0	5.7	2.9	3.6
Feeding habits				
DF—Deposit-feeder (%) [**Acr,Cst,Eri,Bpy**]	31.7	38.9	43.6	25.0
DF_S—Deposit-feeder + scraper (%) [**Bec,Nhi**]	45.7	45.1	41.4	71.6
DF_O—Deposit-feeder + opportunist (%) [**Pfi**]	0.0	5.7	5.7	3.2
P—Predator (%) [**Ave,Mal**]	12.2	5.1	9.1	0.1
G—Grazer (%) [**Mva**]	5.0	5.2	0.2	0.0
Thermal preference				
HE—Highly eurythermal (%) [**Acr,Cst,Ave,Nhi,Mal,Pfi**]	59.0	57.3	58.1	92.3
E—Eurythermal (%) [**Bpy**]	2.2	5.4	3.8	4.8
ME—Moderately sthenothermal (%) [**Mva**]	6.0	6.9	6.4	0.0
S—Stenothermal (%) [**Bec, Eri**]	32.9	30.5	31.7	2.9

Mean values in the semi-lentic (µSL) and lotic (µLR) habitats; mean values in the dammed (µD) and undammed (µUN) states of the Tiber River. Nhi: *Nitocra hibernica*; Acr: *Attheyella (Attheyella) crassa*; Bec: *Bryocamptus (Echinocamptus) echinatus*; Bpy: *Bryocamptus (Bryocamptus) pygmaeus*; Cst: *Canthocamptus (Canthocamptus) staphylinus*; Eri: *Epactophanes richardi*; Ave: *Acanthocyclops vernalis*; Mal: *Macrocyclops albidus*; Mva: *Microcyclops varicans*; Pfi: *Paracyclops fimbriatus*. The assignment of traits to each species is indicated by acronyms in bold in squared brackets. Taxonomic diversity indices: species richness (S), Margalef’s (d), Pielou’s (J), Shannon’s (H), Simpson’s (1-λ). Functional diversity indices: Functional Richness (FRic), Functional Evenness (FEve), Functional Divergence (FDiv), Functional Dispersion (FDis).

**Figure 2:**
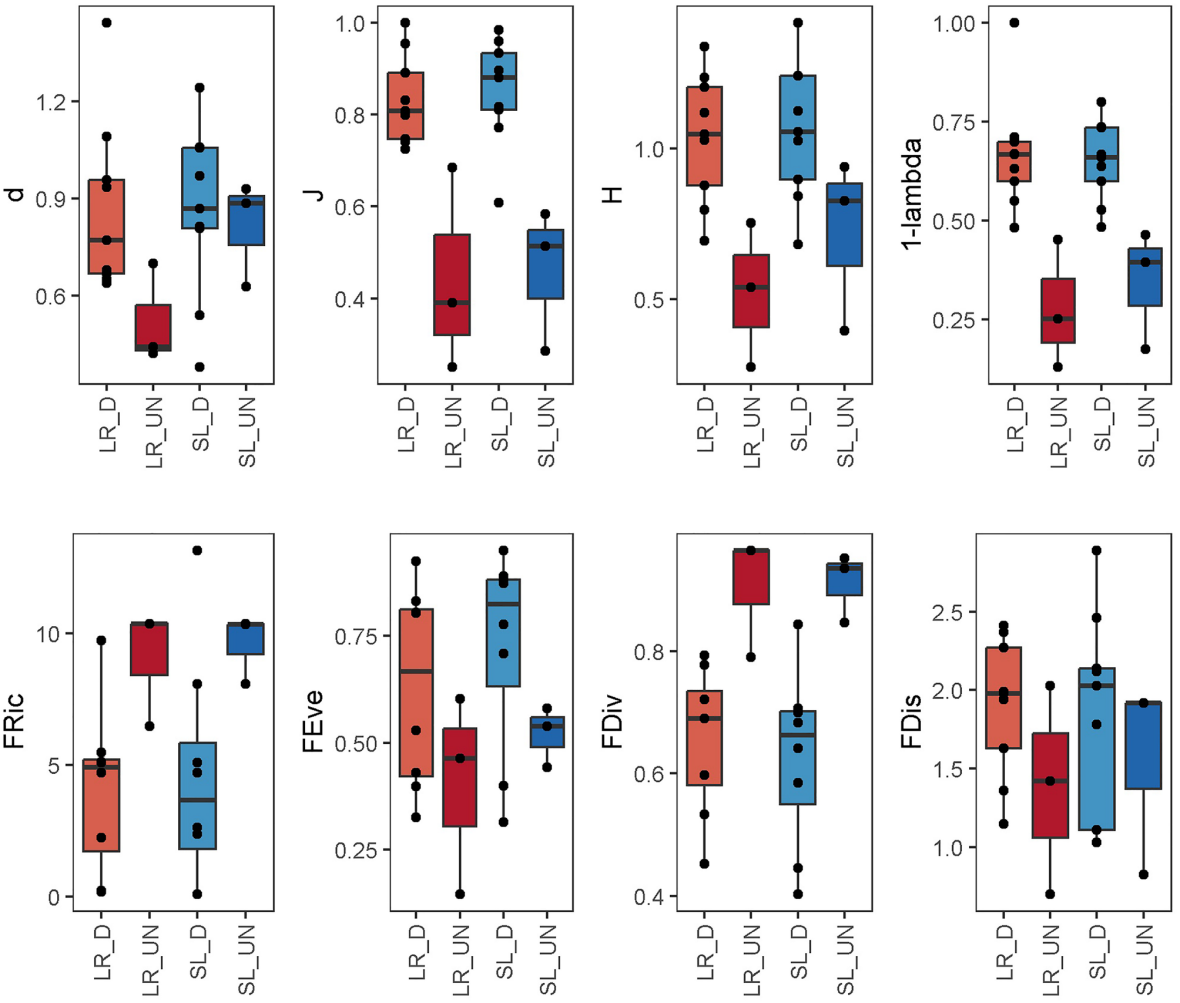
Boxplots illustrating the taxonomic and functional diversity indices. Each boxplot includes median lines, 25–75th percentile boundaries, and whiskers for 10–90th percentiles. Dammed (D) and undammed (UN) states and semi-lentic (SL) and lotic habitats (LR). Margalef’s (*d*), Pielou’s (*J*), Shannon’s (*H*), and Simpson’s (*1-λ*) indices. Functional Richness (*FRic*), Functional Evenness (*FEve*), Functional Divergence (*FDiv*), Functional Dispersion (*FDis*).

The indices of taxonomic diversity varied between the two habitat types and states (Fig. 2). In detail, the Margalef’s (*d*) index was significantly higher in the semi-lentic habitat than in the lotic one (Pseudo-F_1,23_ = 22.17, p-value = 0.033), while Pielou’s (*J*), Shannon’s (*H*) and Simpson’s (*1-λ*) indices were significantly higher in the dammed state than in the undammed one (respectively: Pseudo-F_1,23_ = 73.94, p-value = 0.033; Pseudo-F_1,23_ = 40.20, p-value = 0.005; Pseudo-F_1,23_ = 51.66, p-value = 0.004). The number of species (S) did not show significant variations between the two habitats or states (p-values > 0.05; Table 2). The SIMPER analysis revealed that the species composition between the dammed and undammed state was 65.48% dissimilar. *Nitocra hibernica* was the most contributing species (33%) to the dissimilarity (the species was more abundant in the undammed state; Supplementary Table S3), while *B. echinatus* was more abundant (14%) and occurred more frequently in the dammed state, followed by *A. crassa* (13%) (Supplementary Table S3). The DistLM analyses showed that granulometry explained 38% (R^2^) of the taxonomic composition (AICc = 168.12), where the best model included pebbles and silt (AICc = 168.12; R^2^ = 0.29). Samples containing higher copepod abundances were significantly correlated with higher silt content (Fig. S2). The best linear model built with the chemical and physical parameters indicated chloride as the most influential factor, explaining alone 39% of the taxonomic composition (AICc = 55.03), where abundances of most of the species were significantly correlated with low chloride concentrations (Fig. S2).

### Functional composition

The average values of functional variables in the Tiber River at the sampling station are reported in Table 2. The indices of functional diversity did not vary significantly between the dammed and undammed states nor between the semi-lentic and lotic habitats, except for the functional divergence, which was significantly lower in the dammed state compared to the undammed one (Pseudo-F_1,23_: 10.28, p-value = 0.047; Fig. 2). In addition, the average biomass of copepod samples (Table 2) did not differ between the two habitats (Pseudo-F_1,23_: 0.04, p-value = 0.961). However, it significantly differed between the undammed and dammed states (Pseudo-F_1,23_: 30.07, p-value = 0.017), with a higher average biomass of 71 μg dry C in the undammed state *versus* 22 μg dry C in the dammed one. The composition of the life stages, body shape, locomotion, diet, feeding habits, and thermal tolerance did not significantly differ between the two habitat types nor between the dammed and undammed states (p-values < 0.05).

**Figure 3:**
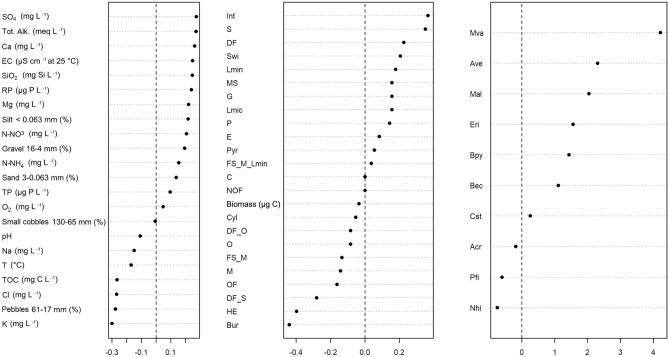
RQL plot. First axis RQL scores for the environmental (left), trait (central), and species abundance (right) data. Abbreviation as in Tables 1 and 2.

The first axis of co-inertia of the RQL analysis explained 82.90% of covariance. Hence, we decided to show and interpret the scores of the first RQL axis only (Fig. 3). The plot delineates a separation of three species (*N. hibernica*, *P. fimbriatus,* and *A. crassa*) from others, highlighting their positive correlation with a range of functional traits encompassing biomass and life history traits such as copepodids, males, and both ovigerous and non-ovigerous females (Fig. 3). All three species are highly eurythermal and burrowers. *Attheyella crassa* and *N. hibernica* have a cylindrical body shape while *P. fimbriatus* is pyriform (Table 2; Fig. 3). Their diet is mainly based on fine sediment and microorganisms, even if *P. fimbriatus* is omnivorous, being deposit-feeders and scrapers (Table 2; Fig. 3). These functional characteristics were, in turn, linked to elevated levels of pebbles, potassium (K), sodium (Na), total organic carbon (TOC), chloride (Cl), temperature (T), and pH (Fig. 3). The plot presented in Fig. 3 further indicates that the other species within the copepod assemblage of the Tiber River at Sansepolcro station were associated with the rest of the traits and environmental variables. Specifically, interstitial species and swimmers were more associated with loosely sorted sediments containing gravel, silt, and sand. Predators and species that consume live microinvertebrates and microphytes, along with grazers, tended to inhabit waters with higher levels of electrical conductivity, phosphates, nitrogenous compounds, and sulfates. However, the fourth-corner analysis indicated that the associations identified by the RLQ analysis, when tested in pairs, did not yield statistically significant relationships (p-values > 0.001).

## Discussion

Beavers are known as ecosystem engineers due to their significant impact on river hydrology, geomorphology, biochemistry, and biological assemblages^8,9^. While a high research effort has been undertaken to assess the effects of beavers on macroinvertebrates and other organisms^40,41^, there has been no research on the beavers’ effects on the meiofauna. The results of this study on the impact of beaver activity on the benthic copepod assemblages of freshwater habitats in Italy are an addition to the growing body of literature on the effects of beavers on aquatic ecosystems.

In this study, the granulometric composition varied substantially between the semi-lentic and lotic habitats at the Sansepolcro sampling station. This finding was consistent with the well-established effect of beaver dams on river morphology and sediment composition observed in other studies^11^. By slowing down or obstructing the river flow, beaver dams act as sediment traps, causing the deposition of finer sediments in the ponds upstream of the dams that would otherwise remain suspended in the water column^42^. This is usually observed in rivers with a medium to high gradient and fast flow, like the headwaters of the Tiber River segment examined in our study^42^. We also observed a substantial decrease in silt content in the undammed state compared to the dammed one, with a corresponding increase in small cobbles and pebbles as also observed in previous studies^9^. Our findings align with previous research^9,43–45^.

The water quality at the Sansepolcro sampling station stood out for its high quality, not affected by habitat types or dammed/undammed states. While beaver dams are known to improve water quality downstream of the dam by trapping pollutants upstream^11,46^, the good water quality at the sampling station was likely due to the low anthropic pressures in the area and the well-preserved and protected riparian zone, which reduces the potential effects of nutrient load. This finding suggests that the recently constructed beaver dam at the Sansepolcro sampling station did not substantially enhance biogeochemical processing in the semi-lentic habitat as also observed in previous studies concerning other young (less than 4/6 years) beaver barriers^46^. Water temperature in the semi-lentic habitat did not significantly differ from that in the lotic one, which is surprising given the known influence of beaver activities on stream temperature dynamics. Beaver dams and foraging behavior can alter river channels, riparian areas, hydrodynamics, and morphology in ways that may affect temperature^47^. The presence of beavers, for example, may lead to reduced shade, increased radiant heating in pond waters, and a consequent temperature rise in ponds compared to lotic reaches, particularly during summer^48^. However, studies have offered conflicting conclusions regarding the thermal impacts of the beaver dam and pond creation^49^, highlighting the need for long-term monitoring to understand the real effects of beavers on water temperature at the Sansepolcro sampling station.

Diving into the taxonomic composition of the copepod assemblage, the diversity indices revealed noticeable taxonomic variation between states, with the dammed state showing a higher taxonomic diversity compared to the undammed state. In addition, the semi-lentic habitat was more diverse than the lotic one. There is a lack of studies on meiofauna to compare our findings. However, in their review, Grudzinski et al.^8^ also reported a mixed/neutral response in nearly half of the studies concerning the effects of beaver dams on aquatic macroinvertebrates. Some studies found higher macroinvertebrate species richness in lotic habitats downstream of the beaver dam compared to ponds, while others found no significant differences in either diversity or biomass^50,51^. The age of the beaver dam at the Sansepolcro sampling station might have played a role, as previous studies have shown that the communities of semi-lentic habitats of newly formed beaver dams are not substantially different from communities of lotic habitats^14^. On the other hand, the functional diversity remained largely consistent across the habitats and states, except for the functional divergence and biomass, which stood out in the undammed state. The lack of difference could be explained by the presence of two openings in the dam. The incomplete barrier might have reduced the degree of habitat heterogeneity or patchiness which are usually created by beavers’ dams or beaver dam analogs^41,52^.

We found that the taxonomic diversity, the biomass, and the functional divergence in the undammed state of the Tiber River were substantially different from the dammed one. In detail, the absence of the beaver dam resulted in a nearly tripled average biomass and a higher functional divergence, while the taxonomic diversity was lower. This finding is in contrast with some previous studies which have shown that beaver engineering does not affect freshwater biodiversity^53^. However, Rosell et al.^43^ already pointed out that the effect of dams built by beavers on riverine ecosystems can vary depending on where these dams are located along the river. According to the analyses, these changes were due to the reduction of silt and the presence of large amounts of pebbles in the undammed state, which improved the habitat availability^54^. The reproductive peak for the species studied usually occurs between July and September^21^; however, we observed a peak in mid-November. Specifically, while the dominant species *N. hibernica* typically reaches its peak abundance in September^55^ an additional increase in abundance in November is unusual for this species. This inconsistency does not support the hypothesis of a seasonal effect. Nonetheless, the flood event that swept away the dam has prevented us from providing conclusive evidence regarding the seasonality of the observed pattern. The disappearance of the beaver dam and the consequent disappearance of the semi-lentic area upstream may have also lowered predation pressure on benthic copepods by macroinvertebrates and fish, leading to positive impacts on copepod productivity and biomass^54,56^. However, we conducted the final sampling survey 45 days after the flood to let the recovery of both prey taxa like copepods and their usual biological controls. On the other hand, the higher functional divergence observed in the undammed state compared with the dammed one indicated a higher degree of niche differentiation in the undammed state. This result suggested that no single trait was overly dominant or rare within the assemblage of the undammed state, pointing towards a more evenly distributed utilization of resources and niche spaces^57^. This observed functional pattern could also be attributed to the Intermediate Disturbance Hypothesis^58^, due to the flood event. Despite conducting our final sampling survey 45 days post-flood, uncertainties persist regarding whether the copepod assemblage fully reverted to its natural state within that time frame.

Granulometry was the main descriptor of the taxonomic composition of benthic copepods at the Sansepolcro station. Accordingly, the grain size is likely the most crucial factor in determining the composition and assemblages of lotic meiofauna^19^. In lotic environments, coarse grain sizes such as pebbles promote higher species richness and abundance compared to areas rich in sand and mud^18,19^, as observed in this study. Our findings align with previous research^9,43–45^. Chloride also appeared to have a moderate negative effect on the copepod abundances except for *N. hibernica*. However, it remains unclear whether this effect is genuine or not since chloride is known to exert negative effects on lotic meiofauna, reducing their diversity and abundance when concentrations exceed 300 mg L^-1^^59^. Importantly, this concentration level does not align with the conditions observed in our study. In addition, the lack of statistical significance in the fourth-corner analysis presents a cautionary note, suggesting that while functional trends are visible, they may not hold across all possible combinations of traits and environmental variables. This could indicate that other unmeasured factors may be influencing these relationships, or that stochastic processes also play a role in the distribution of these species, a complexity often found in ecological studies^39^. It is also a reminder of the inherent challenge in capturing the multi-dimensional interactions that define ecological systems. Critical factors must be addressed, including competition with other meiofaunal taxa and the interplay between organic matter quality and quantity^19^. Finally, the occurrence of two disturbances during the study period—the beaver dam setting and the September 2022 flood event—poses a challenge in accurately reflecting the natural state of the copepod assemblage under undammed conditions. Even with appropriate control, fully disentangling the effects of the flood and its subsequent recovery on copepod assemblages from the potential impacts of the beaver dam is challenging. Consequently, our conclusion cannot be definitive. Nevertheless, considering these challenges, we believe our study represents the best effort possible under the circumstances.

## Conclusions

In this study, we investigated if and how the benthic copepod assemblage of Tiber River responded to a beaver dam that represented the first of its kind in Italy and had a short duration, lasting only a few months. This unique scenario represented an opportunity to observe the potential effects of beaver activity on benthic copepods in the Mediterranean region. We found that the taxonomic composition and functionality of the copepod assemblage remained largely unchanged across the recently formed semi-lentic and lotic habitats. The young age and the incompleteness of the beaver barrier likely reduced the expected effects due to habitat type. On the other hand, substantial differences were evident between the dammed and undammed states, suggesting that beaver dams can have substantial effects on aquatic biodiversity. Our findings highlighted that there is a need for more studies on beaver impact on meiofauna and other small organisms, which are often overlooked despite playing a crucial role in aquatic food webs and biogeochemical cycles. Investigating the effects of beaver dams on benthic copepod assemblages of lotic habitats can contribute to our understanding of the complex interactions between biotic and abiotic factors in freshwater habitats, which can inform conservation and management strategies, including the potential use of beaver reintroduction as a management tool.

### Supplementary Information

Supplementary Information.

## Data Availability

All data generated or analyzed during this study are included in this published article and its Supplementary Information file.
