# Supplementary material for: Effects of a beaver dam on the benthic copepod assemblage of a Mediterranean river
Source: Sci Rep. 2024 Apr 18;14:8956. doi: 10.1038/s41598-024-59456-y (PMC11026539; doi:10.1038/s41598-024-59456-y)

Effects of a beaver dam on the benthic copepod assemblage of a Mediterranean river

Di Lorenzo T. [a,b*], Tabilio Di Camillo A.[a,c], Mori E.[a,b], Viviano A.[a], Mazza G.[a,b,d], Pontalti A.[a], Rogora M.[e], Fiasca B.[c], Di Cicco M.[c], Galassi D.M.P.[c]

[a]National Research Council of Italy, Research Institute on Terrestrial Ecosystems (CN-IRET), Florence, Italy

[b]NBFC (National Biodiversity Future Center), Palermo 90133, Italy

[c]Department of Life, Health and Environmental Sciences, University of L'Aquila, L'Aquila, Italy

[d]CREA Research Centre for Plant Protection and Certification (CREA-DC), Florence, Italy

[e]National Research Council of Italy, Water Research Institute (CNR-IRSA), Verbania Pallanza, Italy

[*]corresponding author tiziana.dilorenzo@cnr.it

Table S1. Granulometric composition (in %) in each sample. State: dammed (D); undammed (UN). RWB: project acronym (Rivers with Beavers), SL: semi-lentic habitat, LR: lotic habitat; P: sample of the semi-lentic habitat; R: sample in the lotic habitat; F22: February 2022, J: June 2022, S: September 2022, N: November 2022.

	State	Boulders (256 mm)	Large cobbles (256-131 mm)	Small cobbles (130-65 mm)	Pebbles (61-17 mm)	Gravel (16-2 mm)	Sand (2 mm)	Silt (<2 mm)
RWB_SL_P1_F22	D	0	0	0	20	70	7	3
RWB_SL_P2_F22	D	0	0	0	20	70	7	3
RWB_SL_P3_F22	D	0	0	0	20	70	7	3
RWB_LR_R1_F22	D	0	0	30	30	37	2	1
RWB_LR_R2_F22	D	0	0	30	30	35	0	5
RWB_LR_R3_J22	D	0	0	30	30	35	0	5
RWB_SL_P1_J22	D	0	0	0	20	63	10	7
RWB_SL_P2_J22	D	0	0	0	20	63	10	7
RWB_SL_P3_J22	D	0	0	0	20	63	10	7
RWB_LR_R1_J22	D	0	0	30	40	25	0	5
RWB_LR_R2_J22	D	0	0	30	60	3	0	7
RWB_LR_R3_J22	D	0	0	30	60	3	0	7
RWB_SL_P1_S22	D	0	0	0	20	58	7	15
RWB_SL_P2_S22	D	0	0	0	20	53	7	10
RWB_SL_P3_S22	D	0	0	0	20	53	7	10
RWB_LR_R1_S22	D	0	0	30	40	20	15	3
RWB_LR_R2_S22	D	0	0	30	57	5	5	3
RWB_LR_R3_S22	D	0	0	30	45	17	5	3
RWB_SL_P1_N22	UN	0	0	0	80	18	2	0
RWB_SL_P2_N22	UN	0	0	0	90	9	1	0
RWB_USL_P3_N22	UN	0	0	0	80	15	4	1
RWB_LR_R1_N22	UN	0	0	30	85	15	0	0
RWB_LR_R2_N22	UN	0	0	30	85	15	0	0
RWB_LR_R3_N22	UN	0	0	30	90	10	0	0

Table S2. Physical and chemical parameters measured in the semi-lentic (RWB_SL) and lotic (RWB_LR) habitats in February 2022 (F22), June 2022 (J22) and September 2002 (S22), when the Tiber River was beaver-dammed and in November 2022 (N22), when it was undammed T: temperature; EC: electrical conductivity at 20 °C; O_2: dissolved oxygen; Tot. Alk: total alkalinity; RP: reactive phosphorous; TP: total phosphorous; Si: silica; TOC: total organic carbon. Labels in bold indicate the undammed state, while those in regular type indicate the dammed state.

		RWB_SL_F22	RWB_LR_F22	RWB_SL_J22	RWB_LR_J22	RWB_SL_S22	RWB_LR_S22	**RWB_SL_N22**	**RWB_LR_N22**
T	° C	8.0	8.0	11.4	11.1	18.4	18.1	14.7	14.7
EC	µS cm^{-1}	399.4	393.9	398.7	399.1	356.1	358.4	363.2	365.9
pH		7.25	8.27	8.26	8.22	8.25	8.26	8.26	8.27
O_2	mg L^{-1}	13.5	12.9	14.4	14.7	12.0	12.7	13.5	13.5
Tot.Alk.	meq L^{-1}	3.822	3.825	3.861	3.842	3.278	3.277	3.327	3.327
Cl	mg L^{-1}	9.5	9.5	9.8	9.8	10.3	10.3	10.2	10.3
SO_4	mg L^{-1}	28.7	29.9	29.8	29.8	29.3	29.3	29.0	28.9
N-NO_3	µg L^{-1}	242	311	242	241	25	25	103	105
N-NH_4	µg L^{-1}	6	16	21	60	11	9	18	10
Ca	mg L^{-1}	61.6	63.0	62.7	63.6	52.3	53.1	54.0	54.0
Mg	mg L^{-1}	13.2	13.3	13.9	12.7	12.5	12.8	12.8	12.0
Na	mg L^{-1}	9.6	10.5	11.3	9.5	10.3	10.4	10.7	10.7
K	mg L^{-1}	1.5	1.7	1.4	1.4	1.65	1.67	1.9	1.9
RP	µg L^{-1}	3	4	4	5	3	3	3	3
TP	µg L^{-1}	10	9	4	5	6	7	6	5
Si	mg L^{-1}	2.37	2.44	2.52	2.52	1.05	1.06	1.35	1.34
TOC	mg L^{-1}	1.73	1.86	1.72	1.93	1.93	1.88	2.00	2.02

Table S3. Abundances of the benthic copepod species, life history (Nau: nauplii; Ofe: ovigerous females; Nof: non- ovigerous females; Juv: copepodids) and percentages of functional traits in each sample collected at Sansepolcro sampling station of the Tiber River. RWB: project acronym (Rivers with Beavers), SL: semi-lentic habitat, LR: lotic habitat, R: sample of the semi-lentic habitat, P: sample if the lotic habitat; F22: February 2022, J: June 2022, S: September 2022, N: November 2022. Cyl: cylindrical; Pyr: pyriform; Bur: burrower; Int: interstitial endobenthic; Swi: swimmer; Fs+M: fine sediment+microorganisms; Fs+M+LMi: fine sediment+microorganisms+living microinvertebrates; Lmi: living microinvertebrates; LMp: living microphytes; Omn: omnivorous; Dfe: deposit feeder; Dfe+Scr: deposit feeder+scraper; Dfe+Opp: deposit feeder+opportunist; Pre: predator; Gra: grazers; HEur: hypereurythermal; Eur: eurythermal; MSte: moderately stenothermal; Ste: stenothermal. Nhi: *Nitocra hibernica hibernica*; Acr: *Attheyella (Attheyella) crassa*; Bec: *Bryocamptus (Echinocamptus) echinatus*; Bpy: *Bryocamptus (Bryocamptus) pygmaeus*; Cst: *Canthocamptus (Canthocamptus) staphylinus*; Eri: *Epactophanes richardi*; Ave: *Acanthocyclops vernalis*; Mal: *Macrocyclops albidus*; Mva: *Microcyclops varicans*; Pfi: *Paracyclops fimbriatus*. Labels in bold indicate the undammed state, while those in regular type indicate the dammed state.

Samples																	Body shape		Locomotion			Diet					Feeding habits					Thermal preference			
	Nhi	Acr	Bec	Bpy	Cst	Eri	Ave	Mal	Mva	Pfi	Nau	Ofe	Nof	Male	Juv	Bio	Cyl	Pyr	Bur	Int	Swi	Fs+M	Fs+M+LMi	LMi	LMp	Omn	Dfe	Dfe+Scr	Dfe+Opp	Pre	Gra	HEur	Eur	MSte	Ste
RWB_SL_P1_F22	0	6	3	0	1	0	0	0	0	0	2	3	2	1	4	7	100,0	0,0	70,0	30,0	0,0	100,0	0,0	0,0	0,0	0,0	70,0	30,0	0,0	0,0	0,0	70,0	0,0	0,0	30,0
RWB_SL_P2_F22	0	6	0	0	0	0	8	0	0	0	1	0	5	3	6	40	42,9	0,0	42,9	0,0	57,1	42,0	0,0	57,0	0,0	0,0	42,0	0,0	0,0	57,0	0,0	100,0	0,0	0,0	0,0
RWB_SL_P3_F22	0	6	4	0	1	0	6	0	0	0	0	3	4	1	4	24	64,7	0,0	41,2	23,5	35,3	64,0	0,0	35,0	0,0	0,0	41,0	23,0	0,0	35,0	0,0	76,5	0,0	0,0	23,5
RWB_RL_R1_F22	5	12	0	0	0	0	2	0	0	0	5	2	3	0	14	10	89,5	0,0	89,5	0,0	10,5	89,0	0,0	10,0	0,0	0,0	63,0	26,0	0,0	10,0	0,0	100,0	0,0	0,0	0,0
RWB_LR_R2_F22	0	3	11	0	3	0	0	6	0	0	1	1	5	4	13	11	73,9	26,1	26,1	47,8	26,1	73,0	26,0	0,0	0,0	0,0	26,0	47,0	0,0	26,0	0,0	52,2	0,0	0,0	47,8
RWB_LR_R3_F22	0	7	4	0	0	0	0	0	9	0	2	1	8	2	9	16	100,0	45,0	35,0	20,0	45,0	87,0	0,0	0,0	12,0	0,0	35,0	20,0	0,0	0,0	45,0	35,0	0,0	45,0	20,0
RWB_SL_P1_J22	1	0	2	0	0	0	0	0	2	0	2	0	1	1	3	4	100,0	40,0	20,0	40,0	40,0	60,0	0,0	0,0	40,0	0,0	0,0	60,0	0,0	0,0	40,0	20,0	0,0	40,0	40,0
RWB_SL_P2_J22	0	10	28	0	0	1	0	0	2	0	0	6	9	12	14	24	100,0	4,9	24,4	70,7	4,9	95,0	0,0	0,0	4,0	0,0	24,0	70,0	0,0	0,0	4,0	24,4	0,0	4,9	70,7
RWB_SL_P3_J22	0	19	15	7	0	0	0	0	0	0	3	1	14	6	20	20	100,0	0,0	46,3	53,7	0,0	100,0	0,0	0,0	0,0	0,0	63,0	36,0	0,0	0,0	0,0	46,3	17,1	0,0	36,6
RWB_LR_R1_J22	0	15	27	0	0	6	0	0	1	0	4	6	18	11	14	31	100,0	2,0	30,6	67,3	2,0	98,0	0,0	0,0	2,0	0,0	30,0	67,0	0,0	0,0	2,0	30,6	0,0	2,0	67,3
RWB_LR_R2_J22	0	7	11	19	0	1	0	1	0	0	0	4	12	13	10	24	97,4	2,6	17,9	79,5	2,6	97,0	2,0	0,0	0,0	0,0	66,0	30,0	0,0	2,0	0,0	20,5	48,7	0,0	30,8
RWB_LR_R3_J22	5	2	16	0	0	0	0	0	0	0	0	4	9	6	4	14	100,0	0,0	30,4	69,6	0,0	100,0	0,0	0,0	0,0	0,0	8,0	91,0	0,0	0,0	0,0	30,4	0,0	0,0	69,6
RWB_SL_P1_S22	13	25	15	0	0	4	0	5	0	0	0	6	27	11	18	33	91,9	8,1	61,3	30,6	8,1	91,0	8,0	0,0	0,0	0,0	40,0	51,0	0,0	8,0	0,0	69,4	0,0	0,0	30,6
RWB_SL_P2_S22	7	14	18	0	0	0	0	1	0	0	2	6	12	2	20	22	97,5	2,5	52,5	45,0	2,5	97,0	2,0	0,0	0,0	0,0	35,0	62,0	0,0	2,0	0,0	55,0	0,0	0,0	45,0
RWB_SL_P3_S22	22	6	12	1	0	0	0	3	0	0	0	7	7	10	20	28	93,2	6,8	63,6	29,5	6,8	92,0	7,0	0,0	0,0	0,0	16,0	76,0	0,0	7,0	0,0	70,5	2,3	0,0	27,3
RWB_LR_R1_S22	14	15	32	0	10	0	0	0	0	1	0	4	30	6	32	51	98,6	1,4	55,6	44,4	0,0	98,0	0,0	0,0	0,0	1,0	34,0	63,0	1,0	0,0	0,0	55,6	0,0	0,0	44,4
RWB_LR_R2_S22	0	1	0	0	0	0	0	0	0	1	0	0	1	1	0	2	50,0	50,0	100,0	0,0	0,0	50,0	0,0	0,0	0,0	50,0	50,0	0,0	50,0	0,0	0,0	100,0	0,0	0,0	0,0
RWB_LR_R3_S22	49	35	9	0	0	0	0	7	0	0	0	9	50	12	29	43	93,0	7,0	84,0	9,0	7,0	93,0	7,0	0,0	0,0	0,0	35,0	58,0	0,0	7,0	0,0	91,0	0,0	0,0	9,0
**RWB_SL_P1_N22**	108	5	0	5	0	0	0	1	0	0	0	1	68	14	35	62	99,2	0,8	95,0	4,2	0,8	99,0	0,0	0,0	0,0	0,0	90,0	8,0	0,0	0,0	0,0	95,8	4,2	0,0	0,0
**RWB_SL_P2_N22**	57	3	2	8	0	0	0	0	0	4	1	1	34	11	28	43	94,6	5,4	86,5	13,5	0,0	94,0	0,0	0,0	0,0	5,0	14,0	79,0	5,0	0,0	0,0	86,5	10,8	0,0	2,7
**RWB_SL_P3_N22**	66	9	11	4	0	0	0	0	0	2	0	2	38	19	33	64	97,8	2,2	83,7	16,3	0,0	97,0	0,0	0,0	0,0	2,0	14,0	83,0	2,0	0,0	0,0	83,7	4,3	0,0	12,0
**RWB_LR_R1_N22**	109	0	0	7	0	0	0	0	0	1	1	2	53	19	44	161	99,1	0,9	94,0	6,0	0,0	99,0	0,0	0,0	0,0	0,0	5,0	93,0	0,0	0,0	0,0	94,0	6,0	0,0	0,0
**RWB_LR_R2_N22**	63	0	2	3	0	0	0	0	0	5	2	2	30	7	34	41	93,2	6,8	93,2	6,8	0,0	87,0	0,0	0,0	0,0	12,0	3,0	90,0	6,0	0,0	0,0	93,2	4,1	0,0	2,7
**RWB_LR_R3_N22**	65	23	0	0	0	0	0	0	0	5	0	2	31	20	40	57	94,6	5,4	100,0	0,0	0,0	95,0	0,0	0,0	0,0	4,0	20,0	74,0	4,0	0,0	0,0	100,0	0,0	0,0	0,0

Figure S1 Trends of the accumulation curves of the observed (Sobs) and expected species richness in the monitored segment of the Tiber River at Sansepolcro sampling station. The results of the species richness estimators were as follows (expressed in species counts): Chao1 = 10; Chao2 = 10; Jacknife1 = 10; Jacknife2 = 10; Bootstrap = 10; MM (Michaelis-Menten) = 10.31, UGE (Unbiased Gradient Estimator) = 10

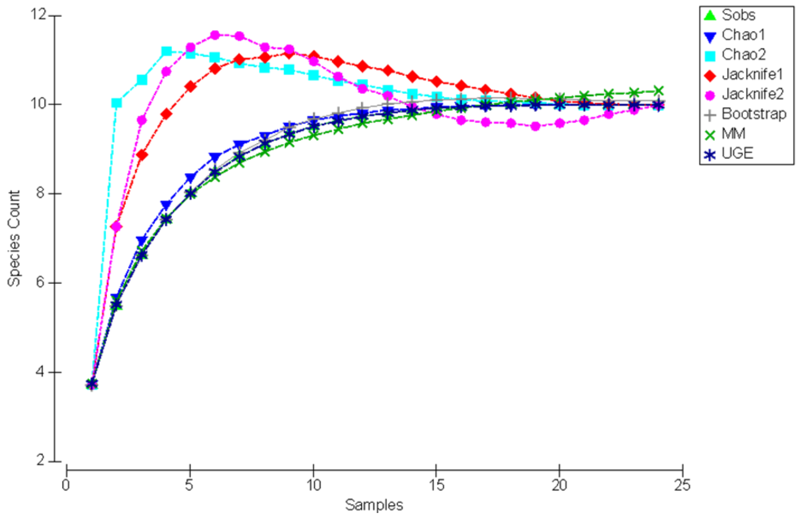

Figure S2 dbRDA plots of the copepod species abundances (the range of abundances per each species are indicated in the left panels) of Sansepolcro station from the parsimonious DistLM models with a) two granulometric variables (pebbles and silt) and b) one chemical-physical variable (chloride). Nhi: *Nitocra hibernica hibernica*; Acr: *Attheyella (Attheyella) crassa*; Bec: *Bryocamptus (Echinocamptus) echinatus*; Bpy: *Bryocamptus (Bryocamptus) pygmaeus*; Cst: *Canthocamptus (Canthocamptus) staphylinus*; Eri: *Epactophanes richardi*; Ave: *Acanthocyclops vernalis*; Mal: *Macrocyclops albidus*; Mva: *Microcyclops varicans*; Pfi: *Paracyclops fimbriatus*.

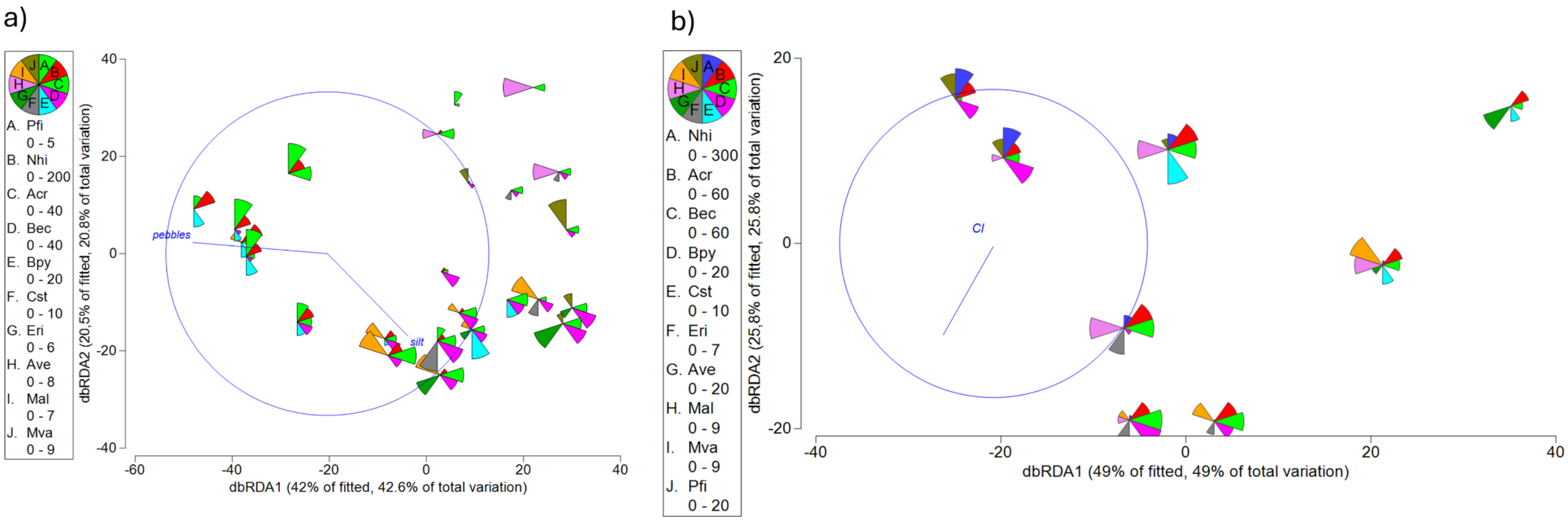

Supplement: Supplementary file 1 — Supplementary Information. [file 41598_2024_59456_MOESM1_ESM.pdf]
